# Serological Evidence of Discrete Spatial Clusters of *Plasmodium falciparum* Parasites

**DOI:** 10.1371/journal.pone.0021711

**Published:** 2011-06-29

**Authors:** Philip Bejon, Louise Turner, Thomas Lavstsen, Gerald Cham, Ally Olotu, Chris J. Drakeley, Marc Lievens, Johan Vekemans, Barbara Savarese, John Lusingu, Lorenz von Seidlein, Peter C. Bull, Kevin Marsh, Thor G. Theander

**Affiliations:** 1 Centre for Geographic Medicine Research, Kenya Medical Research Institute/Wellcome Trust Programme, Coast, Kilifi, Kenya; 2 Centre for Clinical Vaccinology and Tropical Medicine, Nuffield Department of Medicine, University of Oxford, Oxford, United Kingdom; 3 Centre for Medical Parasitology, University of Copenhagen and Copenhagen University Hospital (Rigshospitalet), Copenhagen, Denmark; 4 Department of Immunity and Infection, Faculty of Infectious and Tropical Diseases, London School of Hygiene and Tropical Medicine, London, United Kingdom; 5 GlaxoSmithKline Biologicals, Rixensart, Belgium; 6 Program for Appropriate Technology in Health (PATH) Malaria Vaccine Initiative, Bethesda, Maryland, United States of America; 7 National Institute for Medical Research, Tanga Centre, Tanga, Tanzania; 8 Menzies School of Health Research, Casuarina, Australia; Walter & Eliza Hall Institute, Australia

## Abstract

**Background:**

Malaria transmission may be considered to be homogenous with well-mixed parasite populations (as in the classic Ross/Macdonald models). Marked fine-scale heterogeneity of transmission has been observed in the field (i.e., over a few kilometres), but there are relatively few data on the degree of mixing. Since the *Plasmodium falciparum* Erythrocyte Membrane Protein 1 (PfEMP1) is highly polymorphic, the host's serological responses may be used to infer exposure to parasite sub-populations.

**Methods and Findings:**

We measured the antibody responses to 46 individual PfEMP1 domains at four time points among 450 children in Kenya, and identified distinct spatial clusters of antibody responses to individual domains. 35 domains showed strongly significant sero-clusters at p = 0.001. Individuals within the high transmission hotspot showed the greatest diversity of anti-PfEMP1 responses. Individuals outside the hotspot had a less diverse range of responses, even if as individuals they were at relatively intense exposure.

**Conclusions:**

We infer that antigenically distinct sub-populations of parasites exist on a fine spatial scale in a study area of rural Kenya. Further studies should examine antigenic variation over longer periods of time and in different study areas.

## Introduction

Immunity to the blood stage parasites of *Plasmodium falciparum* malaria is incomplete, and slowly acquired [1]. Exposure to parasites is associated with seroconversion to parasite derived red cell surface antigens, most notably the highly polymorphic PfEMP1 antigens [2], [3]. PfEMP1 antigens mediate cytoadherence of infected red blood cells to host endothelial receptors [4], thereby avoiding circulation through the spleen (where the host may clear parasites), but also causing cerebral malaria [5]. PfEMP1 antigens are encoded by *var* genes, which have been classified into groups, based on their chromosomal location and 5 prime upstream sequence [6], [7]. PfEMP1 antigens contain two main domain types, Cysteine-rich InterDomain Regions (CIDR), and the Duffy Binding-Like (DBL) domains. Domains have very diverse sequences, and PfEMP1 antigens comprise different combinations of between two and nine domains [6], [8].

Spatio-temporal hotspots of malaria transmission have been identified on a fine scale [9], [10], and could become targets for intensified malaria control measures [11], [12], [13]. However, we do not know if there are discrete spatially limited sub-populations of parasites, and so cannot predict the likely outcomes of different targeted control scenarios.

In field studies one may find very focal fine scale heterogeneity of transmission [14], [15], or evidence of widespread dispersion of vectors from known breeding sites [16], [17]. However, neither of these situations directly addresses the mixing of parasites: For instance, it is theoretically possible to have multiple hotspots of high transmission with similar parasite populations transmitting between them, or for what appears to be a single hotspot to have sub-populations of poorly-mixed parasites within it.

Studies have show quite marked variation of parasite genotype over short distances (i.e. kms) in Papua New Guinea [18], [19] and in comparing urban and rural West Africa [20]. However, it is unclear how these findings relate to hotspots of transmission, and whether sub-populations of parasites can be identified on a fine scale in rural Africa.

Seroconversion to the merozoite antigens AMA-1 and MSP1_19_ has been used to identify transmission hotspots [10]. However, antibodies to these antigens do not appear to identify diverse sub-populations, perhaps because these antibodies are highly cross-reactive [21]. Compared with AMA-1 and MSP1_19_, antibodies to PfEMP1 domains are less cross-reactive [22], suggesting that differences in parasite populations might be detectable in the host's variant-specific response. We had access to samples taken from 900 children in Kilifi (Kenya) and Korogwe (Tanzania) who had been enrolled in a randomized controlled trial of the candidate malaria vaccine, RTS,S/AS01E[23]. We measured anti-PfEMP1 antibody responses to 46 different recombinant PfEMP1 domains from 25 different PfEMP1 antigens on multiple plasma samples. We analyse demographic and temporal trends by linear regression and fractional polynomials, respectively. The study was originally designed to examine the impact of vaccination on blood stage immunity. However, the geo-spatial coordinates of homestead location was available in Kilifi, allowing us to identify spatial clusters of serological responses to particular PfEMP1 domains by calculating the Scan statistic [24]. We took account of the repeated measures by clustering the analyses by individual [25], and adjusting by time and age as fixed effects. Details of the domains examined can be found in Table S1.

## Results

### Characteristics of the study area

The study was carried out in two sites, recruiting similar numbers of children. In Kilifi, Kenya, children were recruited in two administrative locations (Pingilikani and Junju), within the Chonyi area in the southern part of Kilifi District. In Tanzania, children were recruited from the catchment areas of three dispensaries (Ngombezi, Mbagai and Makuyuni) in Korogwe district, Tanga Region. Both sites are malaria endemic, with all year round transmission and two high transmission seasons [26]. The transmission intensity has previously been measured as 22–53 infective bites per year in Junju, Kilifi and 90 bites per year in Korogwe [27], [28], although the present transmission intensity is probably much lower [29], [30]. There are successful ITN distribution programmes in both countries [31], [32], and artemether/lumefantrine was the first line anti-malarial treatment. Both areas are rural, and most of the population are subsistence farmers.

### Antibody levels

**Figure 1:**
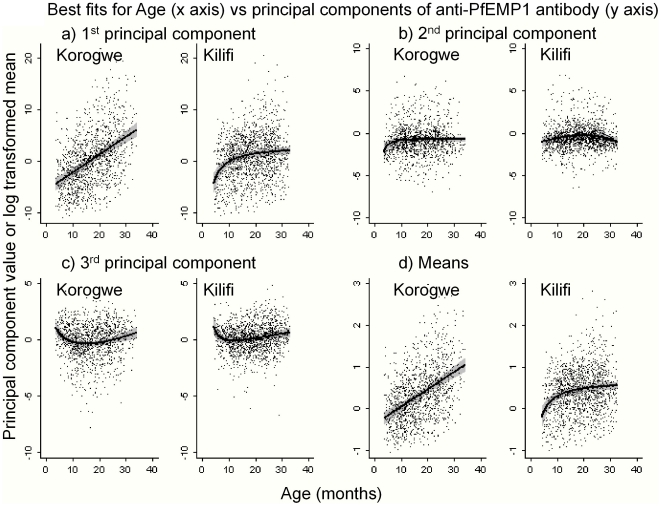
The best fit lines for age vs principal components (panels a–c) and overall mean anti-PfEMP1 antibody responses (panel d) for Korogwe and Kilifi (adjusted by calendar date). The shaded area indicates 95% confidence intervals, and the dots indicate individual raw data points.

The positive control (tetanus toxoid) and negative control (BSA) antigens gave geometric mean ELISA scores of was 32.4 (95%CI 29.3–35.9) and 0.120 (95%CI 0.115–0.123), respectively. Individual PfEMP1 domains had a wide range of geometric mean titers from 0.171 (95%CI 0.163–0.179) for domain 43 (a CIDR domain from a type C PfEMP1) to 16.3 (95%CI 15.4–17.2) for domain 36 (a DBL domain from a type BA PfEMP1). Details of the domains examined can be found in Table S1, and mean antibody responses are shown graphically in figure 1.

### Confirmation of expected associations between antibody responses and age

Age was associated with an average rise in antibody responses of 2.3 fold (95%CI 2.2–2.5 fold) over the interquartile age range of 11 to 22 months. This was strongly significant for 30 domains (p<1×10^−10^) and significant at p<0.001 for a further 44 domains. Domain 46 shows steeply falling antibody responses with increasing age. This domain is part of VAR2CSA [33], which is expressed only during placental infection. Thus, antibodies are acquired in passively transferred maternal antibody and subsequently decay. Tetanus vaccine is given at 1 to 3 months of age, and we observed a 0.9 fold fall in anti-tetanus toxoid responses over the age range tested, i.e. 5 to 34 months (p = 1×10^−11^). No significant variation with age was seen for bovine serum albumin (1.003 fold increase, p = 0.94). The best fit age profiles of individual antibody responses are shown in Figure S1 (adjusted for calendar date). Best-fit lines for domains 5, 8, 9 and 10 (and other domains) in Korogwe show waning antibody in young children followed by increasing production in older children. In contrast, best fits for domains 1, 6, 7, 8, 9 and 10 (and others) in Kilifi indicate rapid acquisition of antibodies in young children followed by a saturation of response in older children.

### Principal component analysis

To reduce the complexity of analysis, we derived three principal components from the 46 PfEMP1 domains. The 1^st^ principal component showed strong positive correlations with all domain antibodies (mean r>0.4) and explained 48% of the variability of the data set. The 2^nd^ principal component explained a further 6% of the variability and was most strongly positively correlated with response to individual CIDR domains (mean correlation 0.12, 95%CI 0.06–0.19) and not strongly negatively or positively correlated with response to DBL domains (mean correlation −0.03, 95%CI −0.08 to 0.01). The 3^rd^ principal component explained 4% of the variability with no trend of correlations with domains of a particular type. Subsequent components explained <3%, were no more informative than responses to a single domain, and so were discarded.

We were concerned that the first principal component might simply reflect exposure to malaria per se. The principal component analysis was re-run including antibody responses to four merozoite antigens (AMA1, EBA-175, MSP1-42 and MSP3), to test whether anti-PfEMP1 domain antibodies and merozoite antibodies would fall into different principal components. In this combined analysis, the first principal component correlated highly with the first principal component of the anti-PfEMP1 only analysis (r = 0.99), but did not correlate with any of the antibodies to merozoite antigens (r = 0.01, 0.01, 0.02 and 0.01, respectively). The 2^nd^ and 3^rd^ principal components from the combined analysis correlated with the merozoite antigens (range r = 0.3 to 0.35), but not to anti-PfEMP1 domain responses. Hence, antibodies to merozoite antigens contributed to different principal components compared with antibodies to PfEMP1 domains. We took the anti-PfEMP1 only principal components forward for further analysis.

### Principal components and age

The anti-PfEMP1 principal components captured the age-profile patterns seen with individual anti-domain response. Increasing age was associated with acquiring anti-PfEMP1 domain antibodies in both Kilifi and Korogwe, as shown by the 1^st^ principal component and by the overall means (Figure 1). In Korogwe the rate of acquisition was constant over the age range tested (5–34 months), but in Kilifi antibody responses appeared to “saturate” past 10 months, and the curve became less steep. In contrast, the curve plotted by the 2^nd^ principal component against age showed an initial steep increase in children below 10 months in Korogwe, and then became flat. In Kilifi, the 2^nd^ component formed a shallow biphasic curve. The 3^rd^ principal component was also biphasic, with a rapid change in children below 10 months of age (during the time of decaying maternal antibody), followed by a more gradual reversal of this change in older children. Date profiles for individual domains and principal components are shown in Figures S2 and S3.

**Figure 2:**
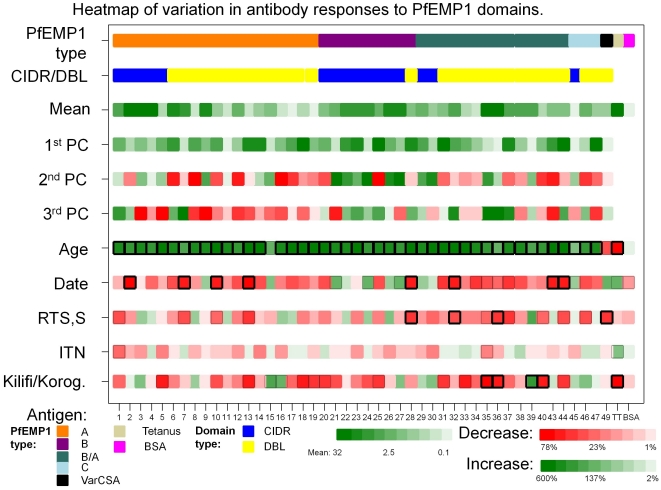
Heatmap of variation in antibody responses to 46 different PfEMP1 domains. The PfEMP1 category and domain category are shown by a colour code in the first two rows (see key at bottom of figure). The third row shows the intensity of mean antibody responses. The positive control (tetanus toxoid) and negative control (BSA) antigens gave geometric mean ELISA scores of 32.4 (95%CI 29.3–35.9) and 0.120 (95%CI 0.115–0.123), respectively. Individual PfEMP1 domains had a wide range of geometric mean response from 0.171 (95%CI 0.163–0.179) for domain 43 (a CIDR domain from a type C PfEMP1) to 16.3 (95%CI 15.4–17.2) for domain 36 (a DBL domain from a type BA PfEMP1). The 4^th^–6^th^ rows show each domains contribution to a principal component analysis. The effect size and significance of the following independent variables is shown; Age, Date, Vaccination with control vaccine vs RTS,S/AS01_E_, ITN use, episodes of malaria and site (Korogwe vs Kilifi). Green shading indicates a positive effect on antibody responses, red shading indicates a negative effect. The thick black boxing indicates p<0.001, thin black boxing indicated p<0.05. The effect size for continuous variables (age and date) is scaled so that the inter-quartile range is comparable to a single category.

The principal components did not vary by insecticide treated bednet (ITN) use, and RTS,S/AS01_E_ vaccination was associated with reduction in the first principal component of borderline significance (Table S2). This corresponded with the analysis of individual anti-PfEMP1 domains: ITN use was associated with small, non-significant reductions in antibody responses (p<0.05 for 2 domains) and vaccination with RTS,S/AS01_E_ was associated with significantly lower responses for 4 individual domains at p<0.001 and a further 13 domains at p<0.05 (the effect size ranging from 0.01 fold to 0.5 fold decreases). The mean antibody levels, principal component analysis, and effects of age, date, ITN use and vaccination are summarized in the heat map in figure 2.

### Geographical clustering of antibody responses

We found fine scale geographical clustering of the serological responses in Kilifi, where children's residences were distributed evenly over a 22×10 km area. In Korogwe, residences were in close knit groups along two lengths of road, and so were not examined further for fine scale geographic variation. SaTScan software was used to identify clusters of high anti-PfEMP1 domain antibody responses using the normal distribution scan[24]. We have previously used SaTScan to identify groups of homesteads at higher transmission intensity, for which we used the term “hotspot”[9]. Anti-PfEMP1 antibody responses do not necessarily indicate transmission intensity. We therefore refer to “clustering” of particular anti-PfEMP1 antibody responses and “hotspots” of indicators of transmission intensity (i.e. malaria incidence and anti-AMA1 antibody responses).

**Figure 3:**
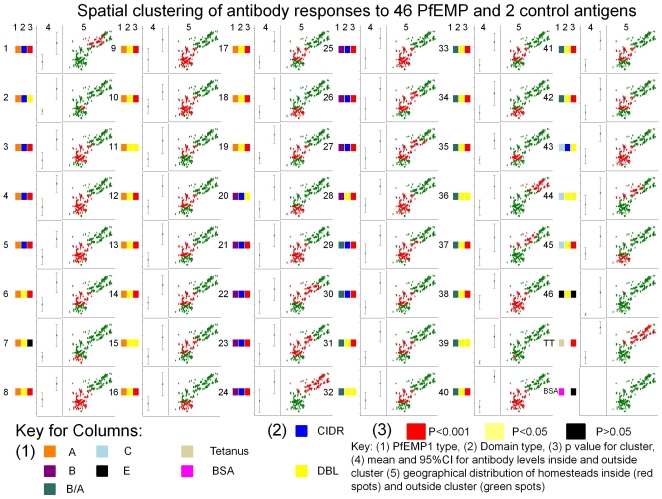
Scatter plots of anti-PfEMP1 domain specific sero-clusters are shown by homestead residence in Kilifi only (longitude vs latitude). The mean responses and 95% confidence intervals inside and outside the hotspot are shown on a log scale to the left of each scatter plot. Red or green shading shows location inside or outside the hotspot, respectively. For example, domain number 1 (top left panel) is from a group A PfEMP1 (1, orange), is a CIDR domain (2, blue), and shows statistically significant clustering at the p = 0.001 level (3, red), antibody responses within the cluster are well above the 95% CI for responses outside (boxed plot of two points with confidence intervals), and on an aerial view the hotspot is in the North-east of the study area (indicated by the red spots).

When analysed individually, 35 domains showed strongly significant sero-clusters at p = 0.001 (Figure 3). A further nine showed significant sero-clusters at p<0.05, and only two domains (numbers 7 and 46, the pregnancy associated VAR2CSA) did not cluster significantly. 20 of the 44 significant sero-clusters grouped in the south-west part of the study area. However, four clusters (i.e. for domains 16, 18, 20 and 34) were to the south of this focus, and five domains (i.e. 1, 24, 36, 37 and 44) were to the north of this focus. Ten domains formed sero-clusters large enough to include both the south-west and the southern area. Every homestead in which a child was sampled was included in at least one cluster. Anti-bovine serum albumin (BSA) antibody responses did not significantly cluster. Tetanus toxoid antibody responses formed a cluster in the north-east. The largest dispensary is located in the center of this cluster, and is the chief source of anti-tetanus vaccination in the study area.

**Figure 4:**
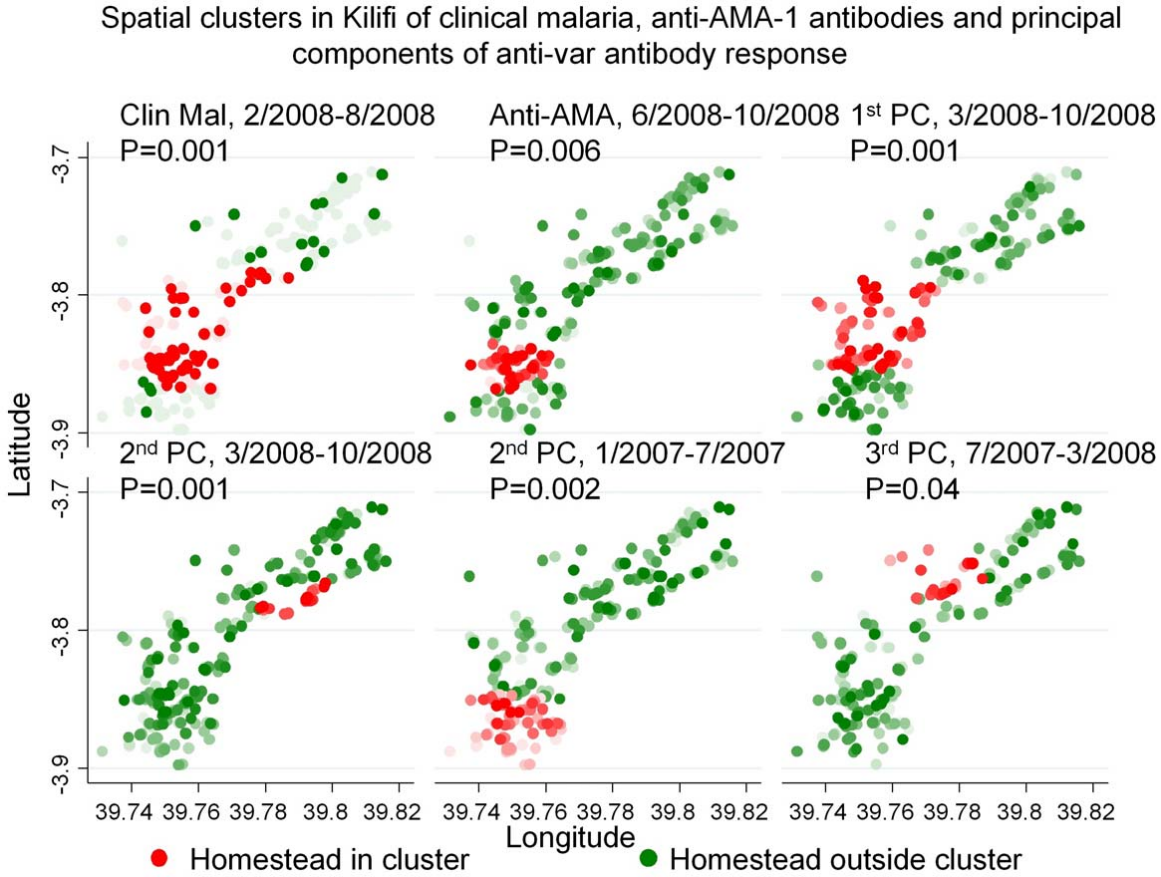
Hotspots for clinical malaria episodes, anti-AMA-1 antibodies and for the first 3 principal components of antibody responses to 46 different PfEMP1 domains are shown in Kilifi. The intensity of the red or green spots indicates the range of the principal component, but red or green shading depends on being inside or outside the hotspot, respectively. The time period during which each sero-cluster was present and the statistical significance of the scan statistic is indicated above each panel.

To more readily visualize these patterns, we plotted spatial clusters based on the three principal components of anti-PfEMP1 responses. The hotspot of transmission indicated by clinical malaria episodes (p = 0.001) and antibody responses to the merozoite antigen AMA-1 (p = 0.006) coincided with the 1^st^ principal component cluster for anti-PfEMP1 domain responses (p = 0.001) in the south-west of the study area between Feb/March and August 2008 (figure 4). The 2^nd^ principal component formed a concurrent cluster of positive values 7 kilometres to the North-east (p = 0.001), and an earlier cluster of negative values (Jan–June 2007) just to the South (p = 0.002). The 3^rd^ principal component formed an earlier cluster (July 2007–Mar 2008) four kilometres to the North-east (p = 0.04).

### Heterogeneity of exposure by individual within hotspots

Some children living within the high transmission hotspot did not experience an episode of malaria and had low AMA-1 antibody responses. Conversely, some children outside the hotspot had episodes of malaria and high AMA-1 antibody responses. Hence, although geographically the 1^st^ principal component sero-cluster overlapped the high transmission hotspot (Figure 4, Table S3), when analysed by individual the 1^st^ principal component did not correlate with transmission intensity (Table S4). This can be seen graphically by comparing the intensity of shading of homesteads between the panels of figure 4 Despite the 1^st^ component sero-cluster overlapping the transmission hotspots (indicated by AMA-1 antibody and malaria episodes), the individuals at highest exposure intensity (i.e. highest AMA-1 antibody responses or malaria episodes) did not necessarily have high anti-PfEMP1 1^st^ principal component values.

In order to examine the diversity of anti-PfEMP1 domain responses, we calculated the variance of anti-PfEMP1 domain responses for each individual (i.e. low variance indicates similar responses by that individual to a range of domains, high variance indicates a range of high and low responses). The individual variances decreased with age, but increased with markers of recent intensity of exposure such as AMA-1 antibody responses or a positive malaria slide (Table S5). This implies that over the long term exposure results in an accumulation of diverse responses to anti-PfEMP1 domains, but in the short term, intense exposure skews the response towards a limited number of domains. However, in the 1^st^ principal component hotspot (where transmission was high) the variance of anti-PfEMP1 responses was reduced (i.e. the high responses in the hotspot were not skewed to particular domains).

## Discussion

We have demonstrated that the host response to polymorphic parasite antigen (PfEMP1) is spatially clustered at a fine scale. This was demonstrated using the responses to 46 different individual PfEMP1 domains, and also using the first three principal components of these responses.

The hotspot of transmission coincided with the 1^st^ principal component cluster for anti-PfEMP1 domain responses, but the 2^nd^ and 3^rd^ principal components clustered outside the transmission hotspot. Although geographically the 1^st^ principal component sero-cluster overlapped the high transmission hotspot (Figure 4), at the level of individual, the 1^st^ principal component did not correlate with transmission intensity (Table S4).

Taking these findings together with the different associations of the principal components at the individual and geographical levels we suggest the following explanation: Hotspots of exposure include the greatest circulating parasite population, and therefore the greatest diversity of PfEMP1 expression. Outside the hotspot, fewer parasites are circulating and therefore diversity is lower. However, at the individual level, intensity of exposure and diversity of parasites within that exposure are different things. For instance, a child living in a low-incidence homestead within the hotspot may be exposed to few infections containing very diverse parasites. On the other hand, a child living in a high-incidence homestead outside the hotspot might be heavily exposed to a less diverse parasite population.

As expected, there were also variations in antibody responses by age. Best-fit lines for domains 5, 8, 9 and 10 (and other domains) in Korogwe are taken to indicate waning maternal antibody in young children followed by increasing endogenous production in older children. This is consistent with the historically very high transmission intensity in Korogwe resulting in higher maternal antibody levels [34]. In contrast, best fits for domains 1, 6, 7, 8, 9 and 10 (and others) in Kilifi indicate rapid acquisition of antibodies in young children followed by a saturation of response in older children, consistent with the higher transmission intensity in Kilifi compared with Korogwe during the period of study [23]. Domain 46 shows steeply falling antibody responses with increasing age. This domain is part of VAR2CSA[33], which is expressed only during placental infection. Thus, antibodies are acquired in passively transferred maternal antibody and subsequently decay.

What are the caveats to these data? Firstly, we tested PfEMP1 domains based on the 3D7 of the FCR3 falciparum isolates, which may not necessarily reflect those circulating locally in Kenya or Tanzania. However, antibody acquisition was age dependent and associated with parasite exposure, suggesting that there is detectable cross-reactivity between the recombinant domains and those that were actually present in the population. Secondly, principal components cannot be assumed to have underlying biological significance and should not be over-interpreted [35]. However, all the inferences we make can be supported by the results of individual anti-PfEMP1 domain responses, and the principal components are presented as a more accessible summary of the individual domain associations. Finally, our original aim was to examine the effect of vaccination on anti-PfEMP1 recognition (which turned out to be minor) and the study of spatial clusters was opportunistic. The risk of a spurious finding rises on secondary analysis. However, the spatial clustering was strongly significant (p = 0.001 for 35 of 46 domains and p<0.05 for 9 of the remaining 11 domains), so a type I error is unlikely.

The discrete clusters of serological responses are unlikely to be explained by the host. The study population comprises a single ethnic group who depend on subsistence farming throughout. Furthermore, the sero-clusters were limited in time as well as space, despite repeated sampling of the same individuals for the duration of the study.

The discrete sero-clusters more likely indicate diverse sub-populations of circulating parasites. This could result from selection pressure as a result of geographic mosaicism, (i.e. interspersed ecosystems with different selection pressure)[36], or simply founder effects in poorly mixed sub-populations. We have previously shown that the environmental factors that determine transmission hotspots can be detected by the MODIS sensor on board two NASA satellites [9], [37]. We re-confirmed the association between MODIS data and transmission intensity in our present study (Table S6), but we did not find that MODIS data predicted the anti-PfEMP1 domain antibody principal components. Furthermore, *var* genes interact with host immunity and influence pathogenicity [38], [39], [40], and an ecological selection pressure on *var* genes seems unlikely. It is possible that very high linkage disequilibrium might cause an apparent ecological selection, but this could only influence a minority of *var* genes[41]. Taken together, we conclude that host responses are spatially heterogeneous because of founder effects among different sub- populations of parasites [42].

The emergence of sub-populations of parasites is likely to depend on transmission intensity , the distribution of human populations and vector breeding sites [43], and the dispersion distance of the vector. We demonstrate evidence for discrete sub-populations of parasite in the host serological response in an area of rural Kenya at low to moderate transmission intensity, where the vector has an average dispersion distance of 0.5 km [44].

Targeting control measures on transmission clusters of infectious disease may be viewed simply as a means of targeting resources on those who most need it. However, if done on a sufficiently fine scale, it will benefit the wider community by reducing overall transmission intensity [43], [45]. Our data suggest that, in addition, such targeted interventions may reduce the diversity of the circulating parasite population, and thus be of even greater benefit than first thought.

## Methods

### Study design

Antibody titers were measured using samples from a randomized, controlled trial designed to evaluate the efficacy and safety of RTS,S/AS01_E_ against clinical malaria episodes by *P. falciparum* infection (ClinicalTrials.gov number, NCT00380393). The study was conducted in Kilifi, Kenya and Korogwe, Tanzania.

### Ethics statement

The study and amendments received ethical and scientific approval from Kenyan Medical Research Institute National Ethics Committee, the Oxford Tropical Research Ethics Committee, the London School of Hygiene and Tropical Medicine Ethics committee and the Western Institutional Review Board in Seattle. The study was overseen by an Independent data monitoring committee and local safety monitors, and conducted in accordance with the Helsinki Declaration of 1964 (revised 1996) and Good Clinical Practice guidelines. Only children whose parent(s) or guardian(s) gave written informed consent were enrolled. Approved Swahili or Giriama consent forms were used. Illiterate parents thumb printed the consent form, countersigned by an independent, literate witness. These consenting arrangements were approved by the IRBs listed above.

### Clinical trial

Details have been published previously [23]. Briefly 894, children 5–17 months old were randomized and received either RTS,S/AS01_E_ or rabies vaccine in a 1∶1 ratio according to 0, 1, 2 month schedule. Both vaccines were given intramuscularly at the left deltoid. The double blind phase was completed after an average of 8 months follow up (with the clinic visit in March 2008) [23]. Investigators were unblinded, but parents of study participants remained blind. The single blind phase continued on both sites until 12 months post dose 3, and was then further extended in Kilifi, Kenya until October 2008.

### Monitoring for episodes of clinical malaria

The primary endpoint was a clinical episode of malaria, defined as an axillary temperature greater than or equal to 37.5 degrees Celsius, with a *Plasmodium falciparum* parasitemia greater than 2,500 parasites per µL. Active surveillance was implemented with weekly home visits by fieldworkers to identify febrile children. Passive surveillance was implemented by fieldworkers and in the health facilities in the study area. Unintentionally, the implementation of active case detection was delayed by 3 months in Korogwe, but passive case detection was conducted during this period.

### Blood samples

Blood was taken for serological studies before vaccination, one month post dose 3, then on March 2008 irrespective of the time of recruitment (i.e. between 4 and 10 months post dose 3, mean 8 months), 12 months post dose 3 and in October 2008 (irrespective of time of recruitment, i.e. between 12 and 18 months post dose 3, mean 15 months).

### Protein expression

46 recombinant proteins representing domains present in PfEMP1s encoded by var genes in different parasite genomes were expressed. The domains were chosen to represent PfEMP1s belonging to different groupings (groups A, B/A, B, C and E),domains of different types (α, β, γ, δ, ε or ζ) or belonging to various semi-conserved domain cassette structures (Table S1, [6]). Protein expression was performed essentially as described previously (1,3). Primer pairs were designed to contain restriction enzyme sites and used to amplify DBL-encoding fragments from 3D7 genomic DNA. The digested PCR products were cloned into the Baculovirus vector pAcGP67-A (BD Bioscience), designed to contain a V5 epitope upstream of a histidine tag in the C-terminal end of the construct. The identity of the cloned fragments was verified by sequencing. Linearized Bakpak6 Baculovirus DNA (BD Biosciences Clontech) was co-transfected with pAcGP67-A into Sf9 insect cells for generation of recombinant virus particles. Histidine-tagged proteins secreted into the supernatant of infected High-Five insect cells were purified on Co2+ metal-chelate agarose columns. Eluted products were dialyzed overnight against PBS, and verified by SDS-PAGE and western blotting using anti-V5 antibodies. Based on these assays the purity of the recombinant proteins varied between 79 and 90 percentage.

### Covalent coupling of recombinant PfEMP1 proteins to microspheres

Carboxylated luminex microspheres were covalently coupled with the different PfEMP1 domains according to the manufacturer's protocol. Each protein was coupled to a particular fluorescence-coded microsphere type. In each case, the microspheres (1.25×107/ml) were washed repeatedly in distilled water, activated in NaH2PO4 buffer, reacted with 1-ethyl-3-[3dimethylaminopropyl] carbodiimide hydrochloride and N-hydroxysulfosuccinimide (Pierce Biotechnology, Rockford, IL), washed twice in 2-[N-morpholino] ethanesulfonic acid, and covalently linked to the target protein (100 µg/ml microsphere suspension). Equal volumes of microspheres coupled with individual domains were pooled, mixed, split into single-use aliquots, lyophilized in polypropylene vials, sealed under nitrogen gas and stored at −80°C.

### Luminex analysis of plasma samples

The analysis of plasma domain-specific IgG levels was as described previously (2). In brief, lyophilized microspheres were reconstituted with distilled water immediately prior to use, and diluted 1∶333 in Assay Buffer E (ABE; 0.1% BSA, 0.05% Tween-20, 0.05% sodium azide in PBS, pH 7.4). Aliquots (50 µl) were dispensed into the wells of 1.2 µm filter-bottom 96-well microtiter plates (MSBVS 1210, Millipore) pre-wetted with ABE buffer, and washed three times with ABE using a vacuum manifold (Millipore, USA). Frozen plasma samples were thawed at room temperature, mixed by vortexing, and spun (16,000 x g; 5 min) to remove particulates. Plasma samples were diluted 1∶80 in ABE buffer and 50 µl aliquots added to the microsphere wells. After incubation in the dark on a shaking platform (30sec at 1,100 rpm followed by 30 min at 300 rpm), the plates were washed three times in ABE to remove unbound antibody. Biotinylated anti-human IgG (Sigma) antibody (25 µl/well at 1∶500 dilution) was added to the microspheres, which were incubated and washed as above. This was followed by streptavidin-phycoerythrin (Sigma) (50 µl/well at 1∶500), incubation in the dark with shaking (30 sec at 1,100 rpm followed by 10 min at 300 rpm), and washing as above. Finally, the microspheres were re-suspended in 125 µl of ABE and analyzed on a Luminex 100IS instrument set to read a minimum of 100 microspheres per microsphere region. Antibody levels for each domain were expressed as median fluorescent intensity (MFI). Reactivity to beads coated with a recombinant protein consisting of a stretch of peptides not found in the protein databases and expressing parts of the Baculo vector including the V5 antibody tag and the stretch of Histidines used for purification was also measured and subtracted from the MFI values obtained to the antigen coated beads. Median fluorescence indexes from the luminex bead studies were converted to scores by comparison with previously well-characterized serum samples from endemic and non-endemic area. The final score was log-transformed to produce a normal distribution.

### Regression models

Multivariable linear regression models were constructed of the antibody levels to each domain, with the explanatory variables age (in months), calendar date (in days), ITN use, geographical location (Kilifi vs Korogwe), vaccination status (as an interaction with time-period, with the fixed effects pre vs post vaccination; and RTS,S/AS01E vs Rabies vaccination, and the interaction between the two to represent the examine whether the vaccinated group differed after vaccination only or throughout monitoring). The results of regression models were displayed using a heat map (Figure 2), in which the effect size (i.e. coefficient) was used to determine the density of shading for each square and a boxed outline was used to indicate significance (a thick-lined box for p<0.001 and thin-lined box for p<0.05).

### Fractional polynomials

Fractional Polynomials were used to examine for non-linear effects of age and calendar date, using the “mfp” command in Stata 10™, using a significance level of p = 0.05 to retain the more complex model.

### Interactions between age/date and site

Multivariable linear regression models were constructed as described above with an additional interaction term for age/date*site. Since transmission intensity was different in both sites, the data were normalized by multiplying all antibody levels in Kilifi by a constant such that the overall mean antibody level was similar to that in Korogwe. The models with interaction terms were then re-run on this normalized data.

### Hotspots

The SaTScan software [46] was used to calculate the spatial scan statistic for a normally distributed continuous variable [24]. The spatial scan statistic uses a scanning window that moves across space. For each location and size of the window, the observed and expected result is compared, and the window with the greatest ratio of observed to expected result noted. The statistical significance of this cluster (or “hotspot) is then evaluated taking into account the multiple tests for the many potential cluster locations and sizes evaluated [47]. P values were calculated after 1000 permutations.

### Principal component analysis

Stata 10™ was used to derive the first three orthogonal components from all the anti-PfEMP1 domain antibody levels (i.e. excluding BSA and tetanus toxoid). Variables were transformed to give mean = 0, variance = 1. Principal components were extracted and the first three retained. The unrotated components were retained for further analysis. Repeated measures were used per individual, and so time was used as a fixed effect in all subsequent analyses.

## Supporting Information

Figure S1 The best fits lines for age vs antibody responses for 46 PfEMP1 domains (1–46) and 2 control antigens (tetanus toxoid, TT, and bovine serum albimun, BSA) are shown for the two sites (Korogwe and Kilifi) in columns 1 and 2. The log scaled y axes are re-scaled for each antigen, but consistent between the two sites. The color-coding system indicates the major group classification of parent PfEMP1 (column 3) and domain type (column 4), the range of the y axis (column 5), the significance of the non-linear fits for Kilifi and Korogwe (columns 6 and 7) and the significance of the interaction between age and site (column 8), and the significance of the interaction after normalizing the antibody responses for the different means between sites (column 9). For example, domain number 1 (top left panel) shows steadily increasing antibody responses with age in Korogwe (column 1), a rapid increase with age in younger children followed by a plateau in Kilifi (column 2), is from a group A PfEMP1 (column 3, orange), is a CIDR domain (column 4, blue), the range of antibody responses on the y axis cover nearly a 630% increase (column 5, intense blue), a non-linear fit is non-significant for Kilifi (column 6, black), but significant in Korogwe (column 7, intense red), there is a strong interaction between age and site (column 8, intense red), but this interaction is non-significant after normalizing results for transmission intensity (column 9, black).
(TIF)

Figure S2 The best fits lines for date vs antibody response for 46 PfEMP1 domains and two control antigens (tetanus toxoid and BSA) are shown for the two sites (Korogwe and Kilifi) in columns 1 and 2. The y axes are re-scaled for each domain, but consistent between the two sites. The color-coding system indicates the major classification of parent PfEMP1 (column 3) and domain type (column 4), the range of the y axis (column 5), the significance of the non-linear fits for Korogwe and Kilifi (columns 6 and 7) and the significance of the interaction between age and site (column 8), and the significance of the interaction after normalizing the antibody responses for the different means between sites (column 9).
(TIF)

Figure S3 The best fit lines for date vs principal components (panels a–c) and overall mean anti-PfEMP1 antibody responses (panel d) for Korogwe and Kilifi (adjusted by calendar date). The shaded area indicates 95% confidence intervals, and the dots indicate individual raw data points.
(TIF)

Table S1 Details of antigens tested, numbering system, PfEMP1 group and domain class.
(DOCX)

Table S2 Coefficients for the effect of vaccination with RTS,S/AS01_E_ and ITN use on principal components and log transformed mean of anti-PfEMP1 domain antibody responses.
(DOCX)

Table S3 Markers of transmission intensity within clusters determined by principal components of anti-PfEMP1 domain antibody responses.
(DOC)

Table S4 Correlations between anti-PfEMP1 domain antibody response principal components and markers of transmission intensity by individual homestead.
(DOC)

Table S5 Multivariate regression for 1^st^ principal component of anti-PfEMP1 domain antibody response and individual variance of the 46 anti-PfEMP1 domain responses.
(DOC)

Table S6 Correlations between malaria episode incidence, anti-PfEMP1 antibody responses and MODIS satellite data.
(DOC)
