# Supplementary figures and images for: Serological Evidence of Discrete Spatial Clusters of Plasmodium falciparum Parasites
Source: PLoS One. 2011 Jun 29;6(6):e21711. doi: 10.1371/journal.pone.0021711 (PMC3126844; doi:10.1371/journal.pone.0021711)

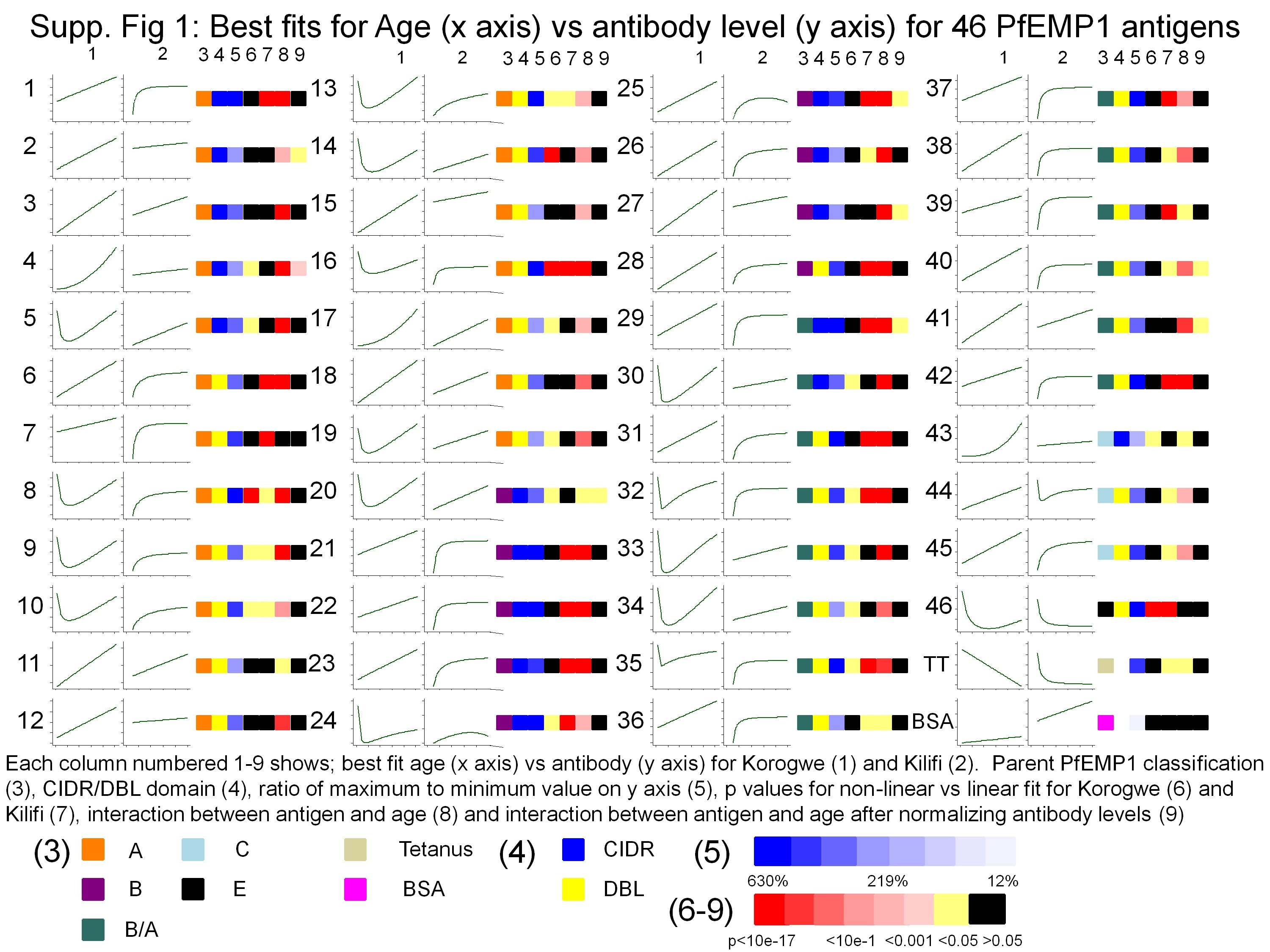

Supplement: Figure S1 — The best fits lines for age vs antibody responses for 46 PfEMP1 domains (1–46) and 2 control antigens (tetanus toxoid, TT, and bovine serum albimun, BSA) are shown for the two sites (Korogwe and Kilifi) in columns 1 and 2. The log scaled y axes are re-scaled for each antigen, but consistent between the two sites. The color-coding system indicates the major group classification of parent PfEMP1 (column 3) and domain type (column 4), the range of the y axis (column 5), the significance of the non-linear fits for Kilifi and Korogwe (columns 6 and 7) and the significance of the interaction between age and site (column 8), and the significance of the interaction after normalizing the antibody responses for the different means between sites (column 9). For example, domain number 1 (top left panel) shows steadily increasing antibody responses with age in Korogwe (column 1), a rapid increase with age in younger children followed by a plateau in Kilifi (column 2), is from a group A PfEMP1 (column 3, orange), is a CIDR domain (column 4, blue), the range of antibody responses on the y axis cover nearly a 630% increase (column 5, intense blue), a non-linear fit is non-significant for Kilifi (column 6, black), but significant in Korogwe (column 7, intense red), there is a strong interaction between age and site (column 8, intense red), but this interaction is non-significant after normalizing results for transmission intensity (column 9, black). (TIF) [file pone.0021711.s001.tif]

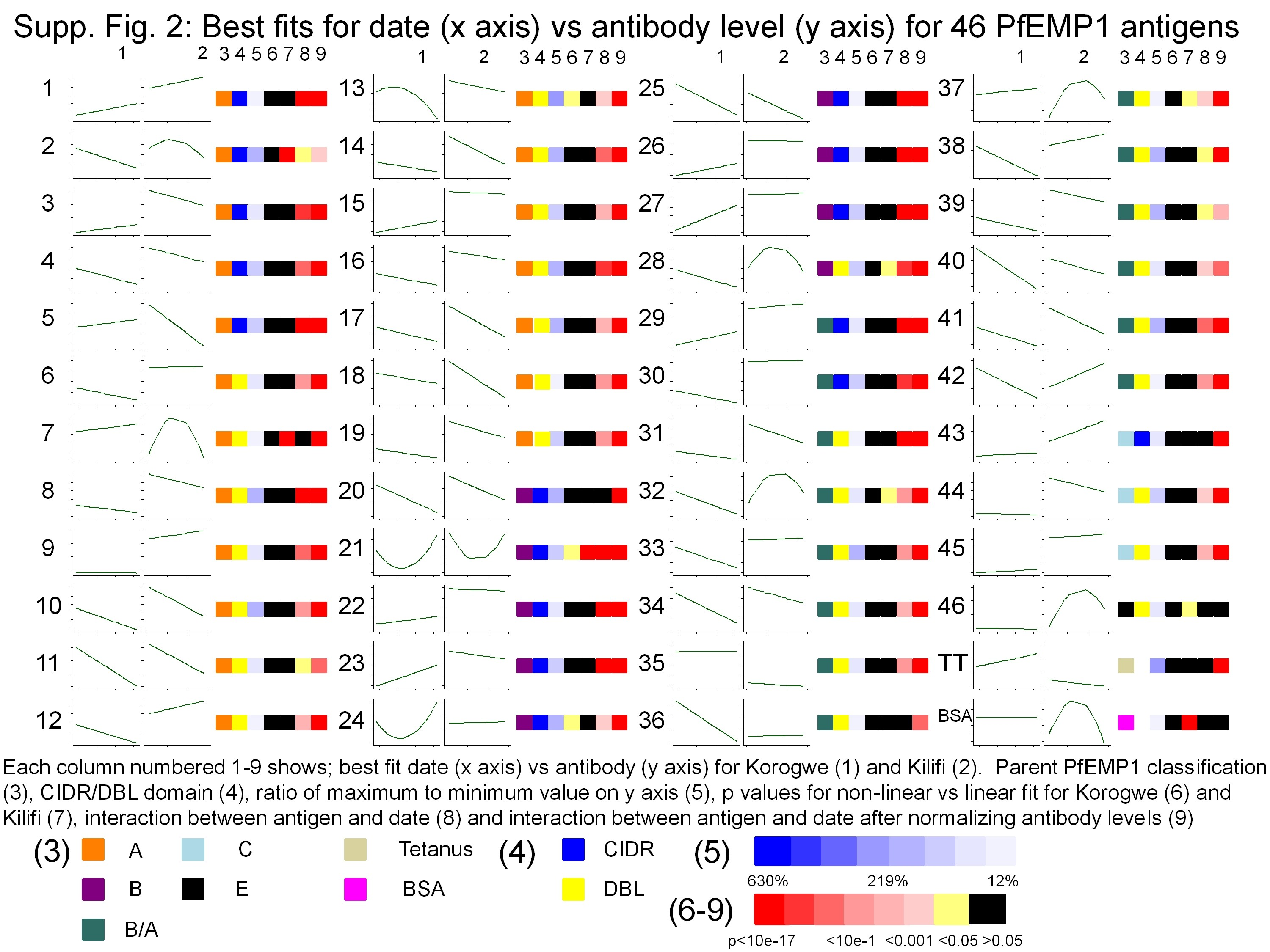

Supplement: Figure S2 — The best fits lines for date vs antibody response for 46 PfEMP1 domains and two control antigens (tetanus toxoid and BSA) are shown for the two sites (Korogwe and Kilifi) in columns 1 and 2. The y axes are re-scaled for each domain, but consistent between the two sites. The color-coding system indicates the major classification of parent PfEMP1 (column 3) and domain type (column 4), the range of the y axis (column 5), the significance of the non-linear fits for Korogwe and Kilifi (columns 6 and 7) and the significance of the interaction between age and site (column 8), and the significance of the interaction after normalizing the antibody responses for the different means between sites (column 9). (TIF) [file pone.0021711.s002.tif]

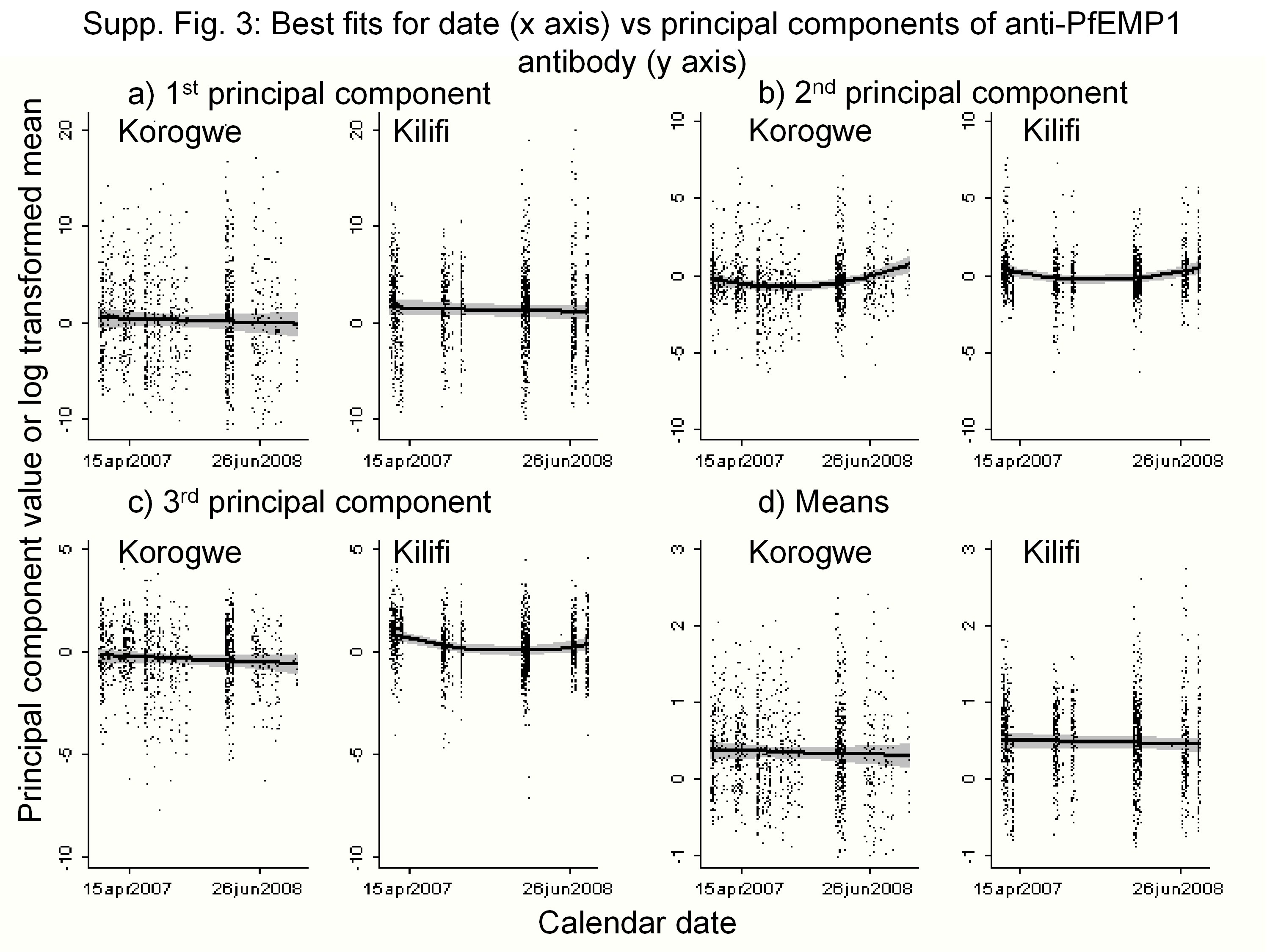

Supplement: Figure S3 — The best fit lines for date vs principal components (panels a–c) and overall mean anti-PfEMP1 antibody responses (panel d) for Korogwe and Kilifi (adjusted by calendar date). The shaded area indicates 95% confidence intervals, and the dots indicate individual raw data points. (TIF) [file pone.0021711.s003.tif]
