# Supplementary material for: Serological Evidence of Discrete Spatial Clusters of Plasmodium falciparum Parasites
Source: PLoS One. 2011 Jun 29;6(6):e21711. doi: 10.1371/journal.pone.0021711 (PMC3126844; doi:10.1371/journal.pone.0021711)
Supplement: Table S1 — Details of antigens tested, numbering system, PfEMP1 group and domain class. (DOCX) [file pone.0021711.s004.docx]

Supplementary Table 1: Domains tested and numbering system.

| UID | Antigen # | PfEMP1 | Genome | Domain class | PfEMP1 group | Domain cassette |
| --- | --- | --- | --- | --- | --- | --- |
| 199200_90 | 1 | IT4var02 | FCR3 | CIDRδ1 | A | 16 |
| 733_92 | 2 | PF13_0003 | 3D7 | CIDRδ1 | A | 16 |
| VAR5CIDR | 3 | PF11_0008 | 3D7 | CIDRβ4 | A | 5 |
| P310_54 | 4 | PFD1235w | 3D7 | CIDRα1.6 | A | 4 |
| GK2_39 | 5 | PF11_0521 | 3D7 | CIDRα1.4 | A | 13 |
| 127128_76 | 6 | PFE1640w | 3D7 | DBLβ1 | A | 1 |
| 129130_67 | 7 | PFE1640w | 3D7 | DBLγ15 | A | 1 |
| P308_47 | 8 | PFD1235w | 3D7 | DBLγ13 | A | na |
| D20-24_42 | 9 | PF11_0008 | 3D7 | DBLγ12 | A | 5 |
| E22-3_55 | 10 | PF11_0008 | 3D7 | DBLδ5 | A | 5 |
| 1314_66 | 11 | PF08_0141 | 3D7 | DBLγ14 | A | 6 |
| 1516_75 | 12 | PF08_0141 | 3D7 | DBLζ5 | A | 6 |
| 2122_77 | 13 | PFD0020c | 3D7 | DBLβ12 | A | 8 |
| 2324_63 | 14 | PFD0020c | 3D7 | DBLγ6 | A | 8 |
| 1112_74 | 15 | PF08_0141 | 3D7 | DBLβ6 | A | na |
| 660C_68 | 16 | PFD1235w | 3D7 | DBLγ13 | A | none |
| 2728_62 | 17 | PFA0015c | 3D7 | full length:  NTSA-DBLα1.3-DBLε8 | A | 3 |
| 2928_85 | 18 | MAL6P1.314 | 3D7 | full length:  NTSA-DBLα1.3-DBLε8 | A | 3 |
| 3028_48 | 19 | PFI1820w | 3D7 | full length:  NTSA-DBLα1.3-DBLε8 | A | 3 |
| 189190_51 | 20 | IT4var21 | FCR3 | CIDRα3.1 | B | na |
| GK25 | 21 | PFC0005w | 3D7 | CIDRα2.4 | B | na |
| GK34_50 | 22 | PFL1955w | 3D7 | CIDRα3.4 | B | 19 |
| SM1112_58 | 23 | PFL1955w | 3D7 | CIDRα3.4 | B | 19 |
| GK28 | 24 | MAL6P1.1 | 3D7 | CIDRα4 | B | na |
| GK17_40 | 25 | PFD0005w | 3D7 | CIDRα2.10 | B | na |
| GK23 | 26 | PFL0005w | 3D7 | CIDRα2.2 | B | na |
| GK37_49 | 27 | PF08_0103 | 3D7 | CIDRα2.2 | B | na |
| 8788_52 | 28 | PFD0005w | 3D7 | DBLδ1 | B | none |
| 6768_89 | 29 | MAL6P1.4 | 3D7 | CIDRγ1 | BA | 43 |
| 4344_53 | 30 | PF08_0140 | 3D7 | CIDRα1.6 | BA | na |
| 155156_65 | 31 | MAL6P1.316 | 3D7 | DBLζ3 | BA | 12 |
| 6162_69 | 32 | MAL6P1.4 | 3D7 | DBLβ5 | BA | 14 |
| 6566_36 | 33 | MAL6P1.4 | 3D7 | DBLδ4 | BA | na |
| 171174_34 | 34 | MAL6P1.316 | 3D7 | DBLγ4 | BA | 6 / 8 |
| 5354_64 | 35 | PFL0020w | 3D7 | DBLζ5 | BA | 6 |
| 5556_43 | 36 | PFL0020w | 3D7 | DBLε4 | BA | 6 |
| 167168_44 | 37 | HB3var22 | HB3 | DBLε2 | BA | 7 |
| 6970_73 | 38 | MAL6P1.4 | 3D7 | DBLε2 | BA | 7 |
| 7172_72 | 39 | MAL6P1.4 | 3D7 | DBLε7 | BA | 7 |
| 7374_38 | 40 | MAL6P1.4 | 3D7 | DBLε3 | BA | 7 |
| 3738_56 | 41 | PF08_0140 | 3D7 | DBLβ12 | BA | 8 |
| 6364_84 | 42 | MAL6P1.4 | 3D7 | DBLγ13 | BA | na |
| GK26_59 | 43 | PFL2665c | 3D7 | CIDRα2.3 | C | na |
| 101102_33 | 44 | PF07_0049 | 3D7 | DBLδ1 | C | na |
| 179180a_46 | 45 | PFD0615c | 3D7 | DBLδ1 | C | na |
| MP319_86 | 46 | IT4var04 | FCR3 | DBLεpam5 | E | 2 |
