# Supplementary material for: Serological Evidence of Discrete Spatial Clusters of Plasmodium falciparum Parasites
Source: PLoS One. 2011 Jun 29;6(6):e21711. doi: 10.1371/journal.pone.0021711 (PMC3126844; doi:10.1371/journal.pone.0021711)
Supplement: Table S2 — Coefficients for the effect of vaccination with RTS,S/AS01E and ITN use on principal components and log transformed mean of anti-PfEMP1 domain antibody responses. (DOCX) [file pone.0021711.s005.docx]

Supplementary Table 2: Coefficients for the effect of vaccination with RTS,S/AS01_E_ and ITN use on principal components and log transformed mean of anti-PfEMP1 domain antibody responses.

| Variable | Coefficient | 5% CI | 95% CI | P |
| --- | --- | --- | --- | --- |
| ITN use |  |  |  |  |
| 1st PC | -0.07 | -0.54 | 0.40 | 0.76 |
| 2nd PC | -0.01 | -0.17 | 0.15 | 0.90 |
| 3rd PC | 0.04 | -0.09 | 0.17 | 0.53 |
| Mean | -0.01 | -0.07 | 0.05 | 0.77 |
| RTS,S/AS01_E_ vaccination | | |  |  |
| 1st PC | -1.01 | 0.07 | -2.08 | 0.07 |
| 2nd PC | 0.12 | 0.48 | -0.24 | 0.51 |
| 3rd PC | 0.14 | 0.44 | -0.16 | 0.37 |
| Mean | -0.12 | 0.01 | -0.25 | 0.06 |

1^st^, 2^nd^ and 3^rd^ PC = 1^st^, 2^nd^ and 3^rd^ principal components of anti-PfEMP1 domain antibody responses. Mean=mean of all anti-PfEMP1 antibody responses (log transformed).
