# Supplementary material for: Serological Evidence of Discrete Spatial Clusters of Plasmodium falciparum Parasites
Source: PLoS One. 2011 Jun 29;6(6):e21711. doi: 10.1371/journal.pone.0021711 (PMC3126844; doi:10.1371/journal.pone.0021711)
Supplement: Table S3 — Markers of transmission intensity within clusters determined by principal components of anti-PfEMP1 domain antibody responses. (DOC) [file pone.0021711.s006.doc]

Supplementary Table 3: Markers of transmission intensity within clusters determined by principal components of anti-PfEMP1 domain antibody responses.

|  | Clinical malaria (episodes per child years of follow up) | | AMA-1 antibody response | | Mean Anti-PfEMP1 response | | Mean Variance of Anti-PfEMP1 response | |
| --- | --- | --- | --- | --- | --- | --- | --- | --- |
| Cluster | Mean | 95%CI | Mean | 95%CI | Mean | 95%CI | Mean | 95%CI |
| Entire study area | 0.55 | 0.5-0.6 | 21.5 | 20-24 | 1.43 | 0.84-2.4 | 25 | 24.6-25.4 |
| 1^st^ PC | 0.99 | 0.7-1.2 | 24.2 | 19-30 | 2.02 | 1.8-2.3 | 22.4 | 21.5-23.3 |
| 2^nd^ PC (NE) | 0.84 | 0.5-1.2 | 26.8 | 19-37 | 1.3 | 1.1-1.5 | 33.9 | 31.4-36.4 |
| 2^nd^ PC (SW) | 0.46 | 0.3-0.6 | 19 | 15-23 | 1.94 | 1.6-2.2 | 22.2 | 20.5-23.8 |
| 3^rd^ PC | 0.29 | 0.1-0.4 | 14.2 | 10-19 | 1.28 | 1.0-1.6 | 43.7 | 32.6-54.7 |

1^st^, 2^nd^ and 3^rd^ PC refers to the clusters determined by the 1^st^, 2^nd^ and 3^rd^ principal components of anti-PfEMP1 antibody responses. NE=North East, SW=South West.
