# Supplementary material for: Serological Evidence of Discrete Spatial Clusters of Plasmodium falciparum Parasites
Source: PLoS One. 2011 Jun 29;6(6):e21711. doi: 10.1371/journal.pone.0021711 (PMC3126844; doi:10.1371/journal.pone.0021711)
Supplement: Table S4 — Correlations between anti-PfEMP1 domain antibody response principal components and markers of transmission intensity by individual homestead. (DOC) [file pone.0021711.s007.doc]

Supplementary Table 4: Correlations between anti-PfEMP1 domain antibody response principal components and markers of transmission intensity by individual homestead.

|  | Clinical malaria | | AMA-1 | |
| --- | --- | --- | --- | --- |
| Cluster | r | P | r | P |
| 1^st^ PC | 0.02 | 0.7 | -0.05 | 0.31 |
| 2^nd^ PC | 0.21 | <0.001 | 0.34 | <0.001 |
| 3^rd^ PC | 0.05 | 0.3 | 0.05 | 0.36 |

1^st^, 2^nd^ and 3^rd^ PC = 1^st^, 2^nd^ and 3^rd^ principal components of anti-PfEMP1 domain antibody responses. Mean=mean of all anti-PfEMP1 antibody responses (log transformed).
