# Supplementary material for: Serological Evidence of Discrete Spatial Clusters of Plasmodium falciparum Parasites
Source: PLoS One. 2011 Jun 29;6(6):e21711. doi: 10.1371/journal.pone.0021711 (PMC3126844; doi:10.1371/journal.pone.0021711)
Supplement: Table S5 — Multivariate regression for 1st principal component of anti-PfEMP1 domain antibody response and individual variance of the 46 anti-PfEMP1 domain responses. (DOC) [file pone.0021711.s008.doc]

Supplementary Table 5: Multivariate regression for 1^st^ principal component of anti-PfEMP1 domain antibody response and individual variance of the 46 anti-PfEMP1 domain responses.

| Variable | Coefficient | 5% CI | 95% CI | p value |
| --- | --- | --- | --- | --- |
| *1st principal component of anti-PfEMP1 responses* | | | | |
| Age | 0.22 | 0.08 | 0.37 | 0.002 |
| Date | -0.003 | -0.02 | 0.01 | 0.74 |
| AMA antibody | 0.03 | -0.6 | 0.6 | 0.9 |
| Kilifi vs Korogwe | -0.8 | -1.9 | 0.31 | 0.16 |
| Blood slide positive | 0.6 | -1.9 | 3.1 | 0.16 |
| Hotspot | 1.33 | 0.52 | 2.1 | 0.001 |
| *Variance of anti-PfEMP1 responses* | | | | |
| Age | -0.008 | -0.01 | -0.00391 | <0.001 |
| Date | 0.00013 | 0 | 0.0003 | 0.043 |
| AMA antibody | 0.091 | 0.05 | 0.12 | <0.001 |
| Kilifi vs Korogwe | -0.1 | -0.16 | -0.03 | 0.003 |
| Blood slide positive | 0.36 | 0.14 | 0.58 | 0.002 |
| Hotspot | -0.13 | -0.18 | -0.07 | <0.001 |
