# Supplementary material for: Serological Evidence of Discrete Spatial Clusters of Plasmodium falciparum Parasites
Source: PLoS One. 2011 Jun 29;6(6):e21711. doi: 10.1371/journal.pone.0021711 (PMC3126844; doi:10.1371/journal.pone.0021711)
Supplement: Table S6 — Correlations between malaria episode incidence, anti-PfEMP1 antibody responses and MODIS satellite data. (DOC) [file pone.0021711.s009.doc]

Supplementary Table 6: Correlations between malaria episode incidence, anti-PfEMP1 antibody responses and MODIS satellite data.

| Principal component of MODIS data | Coefficient | 95% confidence interval | P |
| --- | --- | --- | --- |
| *Correlations with clinical malaria episode incidence* | | | |
| MODIS data 1^st^ PC | 0.099 | 0.038 to 0.16 | 0.002 |
| MODIS data 2^nd^ PC | 0.15 | 0.064 to 0.24 | 0.001 |
| MODIS data 3^rd^ PC | 0.12 | -0.015 to 0.25 | 0.082 |
| *Correlations with 1^st^ principal component of anti-PfEMP1 antibody responses* | | | |
| MODIS data 1^st^ PC | 0.12 | -0.022 to 0.25 | 0.099 |
| MODIS data 2^nd^ PC | 0.17 | -0.047 to 0.38 | 0.12 |
| MODIS data 3^rd^ PC | -0.097 | -0.31 to 0.12 | 0.37 |
| *Correlations with 2^nd^ principal component of anti-PfEMP1 antibody responses* | | | |
| MODIS data 1^st^ PC | -0.052 | -0.12 to 0.015 | 0.13 |
| MODIS data 2^nd^ PC | -0.001 | -0.12 to 0.012 | 0.99 |
| MODIS data 3^rd^ PC | 0.11 | -0.01 to 0.23 | 0.078 |
| *Correlations with 3^rd^ principal component of anti-PfEMP1 antibody responses* | | | |
| MODIS data 1^st^ PC | -0.11 | -0.17 to -0.047 | 0.001 |
| MODIS data 2^nd^ PC | 0.03 | -0.05 to 0.12 | 0.485 |
| MODIS data 3^rd^ PC | 0.088 | -0.003 to 0.18 | 0.06 |

1^st^, 2^nd^ and 3^rd^ PC = 1^st^, 2^nd^ and 3^rd^ principal components.
